# Immortalized human myotonic dystrophy type 1 muscle cell lines to address patient heterogeneity

**DOI:** 10.1016/j.isci.2024.109930

**Published:** 2024-05-07

**Authors:** Judit Núñez-Manchón, Júlia Capó, Alicia Martínez-Piñeiro, Eduard Juanola, Jovan Pesovic, Laura Mosqueira-Martín, Klaudia González-Imaz, Pau Maestre-Mora, Renato Odria, Estefania Cerro-Herreros, Neia Naldaiz-Gastesi, Adolfo López de Munain, Rubén Artero, Dusanka Savic-Pavicevic, Ainara Vallejo-Illarramendi, Kamel Mamchaoui, Anne Bigot, Vincent Mouly, Mònica Suelves, Gisela Nogales-Gadea

**Affiliations:** 1 Grup de REcerca Neuromuscular de BAdalona (GRENBA), Institut d'Investigació en Ciències de la Salut Germans Trias i Pujol (IGTP), Campus Can Ruti, Universitat Autònoma de Barcelona, 08916 Badalona, Spain; 2 Neuromuscular Pathology Unit, Neurology Service, Neuroscience Department, Hospital Universitari Germans Trias i Pujol, 08916 Badalona, Spain; 3 University of Belgrade - Faculty of Biology, Center for Human Molecular Genetics, Belgrade, Serbia; 4 Group of Neurosciences, Department of Pediatrics, UPV/EHU, Hospital Universitario Donostia - IIS Biodonostia, 20014 San Sebastian, Spain; 5 Human Translational Genomics Group. University Research Institute for Biotechnology and Biomedicine (BIOTECMED), Universidad de Valencia, 46100 Burjassot, Valencia, Spain; 6 INCLIVA Biomedical Research Institute, Avenue Menéndez Pelayo 4 acc, 46010 Valencia, Spain; 7 Neurosciences Area, Institute Biodonostia-Department of Neurology, Hospital Universitario Donostia, OSAKIDETZA, an Sebastián, Spain; 8 CIBERNED, CIBER, Instituto Carlos III, Madrid, Spain; 9 Department of Neurosciences. University of the Basque Country, San Sebastian, Spain; 10 Faculty of Health Sciences. University of Deusto, Bilbao-San Sebastian, Spain; 11 Centre for Biomedical Network Research on Rare Diseases (CIBERER), CB23/07/00005, Carlos III Health Institute, 28029 Madrid, Spain; 12 Sorbonne Université, Inserm, Institut de Myologie, Centre de Recherche en Myologie, F-75013 Paris, France

**Keywords:** Health sciences, Biological sciences, Molecular biology, Epigenetics, Cell biology

## Abstract

Historically, cellular models have been used as a tool to study myotonic dystrophy type 1 (DM1) and the validation of therapies in said pathology. However, there is a need for *in vitro* models that represent the clinical heterogeneity observed in patients with DM1 that is lacking in classical models. In this study, we immortalized three DM1 muscle lines derived from patients with different DM1 subtypes and clinical backgrounds and characterized them at the genetic, epigenetic, and molecular levels. All three cell lines display DM1 hallmarks, such as the accumulation of RNA foci, MBNL1 sequestration, splicing alterations, and reduced fusion. In addition, alterations in early myogenic markers, myotube diameter and CTCF1 DNA methylation were also found in DM1 cells. Notably, the new lines show a high level of heterogeneity in both the size of the CTG expansion and the aforementioned molecular alterations. Importantly, these immortalized cells also responded to previously tested therapeutics. Altogether, our results show that these three human DM1 cellular models are suitable to study the pathophysiological heterogeneity of DM1 and to test future therapeutic options.

## Highlights

• Three DM1 immortalized muscle cell models have been generated
• The models reproduce the main DM1 molecular hallmarks
• There is molecular heterogeneity between the three models
• The models are adequate to assess the efficacy of therapies

## Graphical abstract

**Figure:**
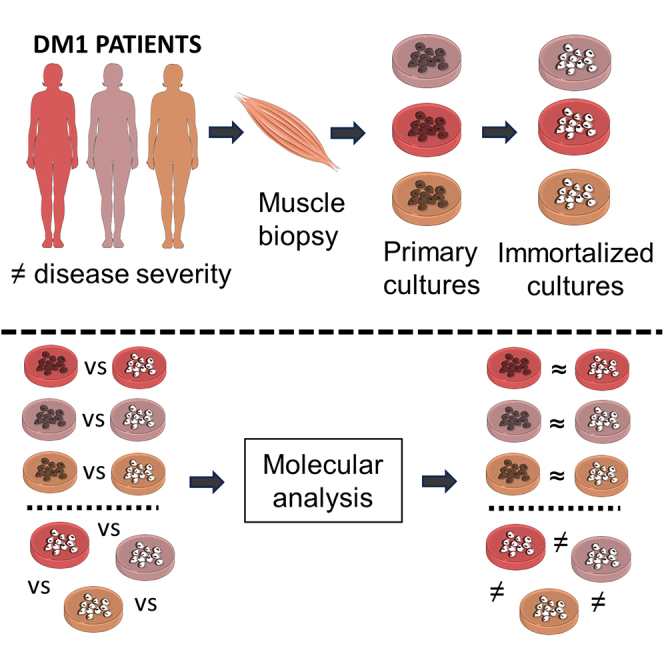

## Introduction

Myotonic dystrophy type 1 (DM1) is an autosomal dominant multisystemic disease caused by a CTG expansion in the 3′ untranslated region of the myotonic dystrophy protein kinase (*DMPK*) gene. It is the most prevalent muscular dystrophy in adults affecting 1 in 8000. It causes a variety of symptoms that include, but are not limited to: muscle weakness, myotonia, cardiac defects, respiratory failure, endocrine alterations such as diabetes, and cognitive impairment. The presence of these symptoms and their severity differs between patients. They can appear at any age and there is an earlier onset of clinical manifestations in successive generations, which is known as genetic anticipation. According to the age of onset, patients with DM1 can be classified as congenital, childhood, juvenile, adult, or late-onset. An earlier age of onset is associated with a greater disease severity and higher CTG expansion sizes.^1^

The CTG expansion length varies in the range between 50 and thousands of repeats^2^ and it has a high degree of instability biased toward expansions in germline and somatic cells. Therefore, the size of the CTG repeat increases over generations in affected pedigrees and through a patients’ life.^3^^,^^4^^,^^5^ Moreover, patients with DM1 present somatic mosaicism in CTG expansion length between different tissues and cell types.^6^^,^^7^ Altogether, this results in patients presenting heterogeneity at the genetic level, which makes it extremely challenging to determine a precise expansion size for each patient.^8^ Currently, CTG expansion size can be determined by small pool PCR which allows researchers to analyze trinucleotide repeat instability^9^ in a quantitative manner, detecting common and low abundant CTG sizes in a pool of cells. This technique allows the characterization of the CTG expansion dynamics of this highly unstable repeat expansion.

Epigenetics may have a role in DM1, as the CTG expansion overlaps with a 3.5 kb CpG island.^10^^,^^11^^,^^12^^,^^13^ The *DMPK* gene is in chromosome 19q surrounded by the *DMWD* (upstream) and *SIX5* (downstream) genes. The *DMPK* locus contains several CpG islands, including the CTCF1 and CTCF2 regions, which are surrounding the CTG expansion. In the case of CTCF1, aberrant methylation patterns have been described by other labs and by ours. For example, DM1 muscle and muscle derived DM1 cells show CTCF1 hypermethylation compared to controls; while blood samples from congenital cases show CTCF1 hypermethylation compared to controls and adult DM1 cases.^12^^,^^14^^,^^15^ However, it is not clear how the hypermethylation of this CpG island contributes to the pathogenesis of the disease, although it has been reported that *DMPK* and the surrounding genes (*DMWD* and *SIX5*) are downregulated in DM1.^16^^,^^17^

It has been demonstrated that RNA gain of function^18^^,^^19^ is the main molecular pathology mechanism in DM1. *DMPK* transcripts containing the CTG expansion accumulate,^20^ form hairpin structures^21^ and agglomerate in the nucleus. These agglomerations receive the name of RNA foci, whose quantity varies between cells, and patient tissues.^20^ We and others have found correlation between RNA foci formation and CTG expansion size, and RNA foci formation and age of onset.^22^^,^^23^ It is known that foci can bind to important cellular proteins, such as the splicing regulator, muscleblind-like 1 (MBNL1).^24^ MBNL1 sequestration and downregulation causes the aberrant splicing of several genes, including insulin receptor (*INSR*),^25^ sarcoplasmic reticulum Ca(2+)-ATPase 1 (*ATP2A1*),^26^ LIM Domain Binding 3 (*LDB3*),^27^ dystrophin (*DMD*),^28^ bridging Integrator 1 (*BIN1*),^29^ and *MBNL1* (which regulates its own splicing).^30^ Some splicing alterations are tightly connected with some of the symptoms that patients with DM1 present: *INSR* is associated with insulin resistance,^31^ *BIN1* with muscle weakness,^32^ *LDB3* with sudden cardiac death^33^ and *ATP2A1* and *DMD* with muscle regeneration.^34^^,^^35^

Cellular DM1 models have proven to be a valuable tool for studying the molecular aspects of diseases and for evaluating the efficacy of potential treatments.^36^^,^^37^ Nowadays, different cellular models have been developed for studying DM1, each with their own pros and cons.^38^ Among them, we have cell lines with artificial expressions of exogenous CTG repeats and patient derived cell lines.^39^ Cells artificially expressing CTGs, even though they reproduce the hallmarks of the disease,^25^^,^^39^ lack the genetic context of the *DMPK* gene, including its regulation by the gene promoter and the expression alterations of adjacent genes (*DMWD* and *SIX5*). This inconvenience is overcome in the patient derived cell lines, which can be either primary or immortalized cells. Primary cells are obtained from patient biopsies. The most frequently used are skin fibroblasts, because they are easier to obtain, and myoblasts, as muscle is one of the main affected tissues in DM1, which can fuse into myotubes. However, primary cultures have limitations in the number and type of experiments that can be performed because they have a reduced number of divisions, and it is ethically controversial to take biopsies from patients with DM1. Since skin biopsies are more accessible, skin fibroblasts can be transdifferentiated into myoblasts by *MYOD1* transduction.^31^ However, it has been shown that the transdifferentiation does not lead to full muscle cell reprogramming.^40^ Another strategy consists in the reprogramming of patient cells into iPSCs.^41^ This approach offers the advantage of differentiating iPSCs into any type of cell, including those located in tissues where biopsies are not feasible, such as cardiomyocytes or neurons which are also affected in DM1.^42^ The downsides of iPSCs include a highly unstable CTG repeat during reprogramming, incomplete cell maturation, and high maintenance cost. To resolve these problems, immortalized muscle patient cells were developed by inserting lentiviral vectors expressing the catalytic subunit of human telomerase (*TERT*), cyclin-dependent kinase 4 (*CDK4*), and cyclin D1 (*CCND1*).^38^ With these changes, cells can divide an unlimited number of times, thus diminishing the need to perform muscle biopsies and allowing the execution of experiments that need a high cell number. Nonetheless, it still needs to be proven that the insertion of these transgenes does not produce alterations in cellular behavior.^43^ To date, there are only three reports in which four myoblast patient derived cell lines have been generated.^44^^,^^45^^,^^46^

In this work, we have immortalized and characterized three DM1 muscle cell lines from patients with adult DM1 that belonged to the juvenile, adult and late onset DM1 subtypes. These patients were clinically heterogeneous in their symptomatology, which is demonstrated by their muscle, heart, and lung pathologies. The immortalized muscle cell models were also heterogeneous in their molecular alterations, but they presented similar alterations to the parental cells from which they were derived. Notably, antisense oligonucleotide treatment rescued a substantial portion of the molecular alterations in these models. In conclusion, the three models generated are adequate models to address the heterogeneity in DM1 and to analyze genetic, epigenetic, transcriptomic, and proteomic alterations, cellular functions, and response to therapies in a more diverse way.

## Results

### Immortalization of muscle cells derived from patients with myotonic dystrophy type 1 with clinical heterogeneity

In this study, we have immortalized myoblasts from 3 patients with DM1 showing different clinical manifestations and degrees of disability (Table 1). An STR variant analysis of 16 locus was performed for line authentication purposes and it confirmed that the immortalized and primary cell lines of each patient shared the same alleles (Table 2). The samples came from three female patients, ranging in age from 36 to 46 at the moment of sampling. Their ages of onset were 15, 27 and 42, placing them in the juvenile, adult, and late onset DM1 subtypes, respectively. Muscular involvement was determined with the Medical Research Council (MCR) scale in both proximal and distal muscles. Patients JCC-DM1 and ADE-DM1 had moderate muscle involvement; they had an MRC score of 4 for proximal muscles and an MRC score of 3 for distal muscles. In contrast, GPM-DM1 had mild involvement, with an MRC score of 5 for proximal muscles and an MRC score of 4 for distal muscles. Cardiac involvement was mild in patients JCC-DM1 and GPM1-DM1, while in ADE-DM1 it was severe. This patient needed a pacemaker after suffering several bouts of syncope and after being diagnosed with an elongated HV interval. Respiratory involvement was mild in ADE-DM1 who had a forced vital capacity (FVC) of 65%, moderate in JCC-DM1 who had an FVC of 58% and severe in GPM-DM1 who had an FVC of 50% and required mechanical ventilation. Finally, to determine their level of functionality, we used the modified Rankin scale (mRS), which determines DM1 patient’s degree of disability and dependence in daily activities. GPM-DM1 had a mRS of 1, implying she did not present significant disability and was able to carry out usual activities. JCC-DM1 had a mRS of 2, implying she suffered a slight disability and even though she could live unassisted, she could not perform all the activities she performed previous to the onset of the disease. ADE-DM1 had a mRS of 4; implying she had a moderately severe disability and wasn’t able to attend bodily functions or walk without assistance. Globally, ADE-DM1 was the most affected patient as she suffered from severe cardiac and functional involvement, as well as moderate muscular involvement. She is followed by JCC-DM1 in severity, who had moderate functional, muscular and respiratory involvement. Lastly, GPM-DM1 had mild involvement in all the parameters studied, except for respiratory function which was severely affected.

**Table 1:** General data and clinical characterization of the patients with DM1 and controls

Sample	Type of sample	Age at sampling	Gender	Age of onset	Muscle involvement	Cardiac involvement	Respiratory involvement	mRS	Type of cellls derived
JCC-DM1	Patient	36	F	15	moderate	mild	moderate	2	Primary and immortalized
GPM-DM1	Patient	46	F	42	mild	mild	severe	1	Primary and immortalized
ADE-DM1	Patient	39	F	27	moderate	severe	mild	4	Primary and immortalized
C7	Control	66	F						Primary
C9	Control	41	M						Primary
C10	Control	26	M						Primary
AB678	Control	53	M						Immortalized
AB1079	Control	38	M						Immortalized
KM1421	Control	13	F						Immortalized

mRS, modified rankin scale.

**Table 2:** STR profiling of the patients from which the cells were isolated and immortalized

Locus	Chromosome location	JCC-DM1	GPM-DM1	ADE-DM1
D8S1179	8	13	10, 14	12, 14
D21S11	21q11.2-21-22	31, 32.2	29, 32.2	30, 33.2
D7S820	7q11.21–22	8, 10	10, 11	10
CSF1PO	5q33.3–34	10, 11	10, 12	11, 13
D3S1358	3p	17, 18	17	16, 17
TH01	11p15.5	6	6	8, 9.3
D13S317	13q22-31	8, 10	11	11
D16S539	16q24-qter	12, 13	12	11
D2S1338	2q35–37.1	17, 19	19, 23	17
D19S433	19q12–13.1	13, 15	12, 13	14, 15
vWA	12p12-pter	14, 16	15, 18	16, 18
TPOX	2p23-2per	9, 11	8, 12	8
D18S51	16q21.3	12, 15	12, 14	14, 15
AMELOGENIN	X: p22.1–22.3 Y: p11.2	X	X	X
D5S818	5q21-31	11, 13	10, 12	11
FGA	4q28	20, 21	25, 26	21, 25

### A high level of CTG repeats instability characterized all studied myotonic dystrophy type 1 cell lines

**Figure 1:**
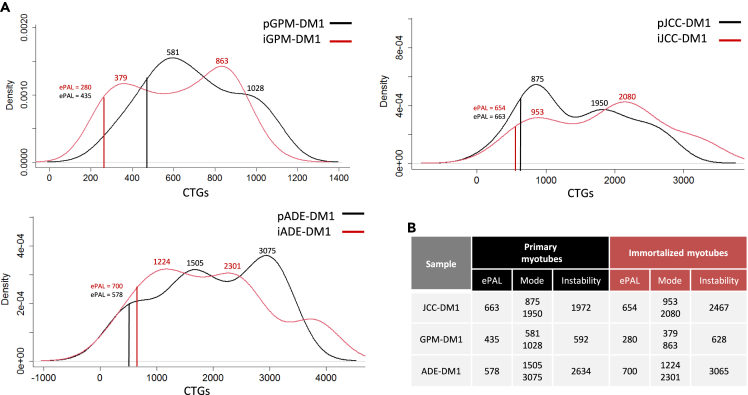
CTG instability is similar between primary and immortalized DM1 cells lines (A) Density plots showing the distribution of the CTG alleles detected in both the primary (black) and immortalized (red) myotubes derived from the three patients participating in the study. We measured between 41 and 105 alleles per sample. (B) CTG expansion ePAL, mode and instability in primary and immortalized myotubes of the three patients with DM1 participating in the study. The progenital allele length was estimated as the 10^th^ percentile of allele frequency distribution. The modal allele length was determined as the most frequent allele. The level of somatic instability was calculated by subtracting the 10^th^ percentile from the 90^th^ percentile. “p” and “i” before cell line name mean primary and immortalized, respectively.

CTG expansion was measured in primary and immortalized cell lines from the three patients with DM1 by small pool PCR, which revealed highly heterogeneous CTG expansion patterns within each patient. We created density plots with the results and calculated the estimated progenitor allele (ePAL), the size of the two main expanded populations, and the expansion instability observed in each cell line (Figures 1A and 11B). Primary GPM-DM1 cells had an ePAL of 435 CTGs and showed two expanded main populations with 581 CTGs and 1028 CTGs, while in the immortalized cells the ePAL was 280 CTGs and the two most abundant populations had 379 and 863 CTGs, respectively. Primary JCC-DM1 cells had an ePAL of 663 CTGs and the average sizes of the two main expansion populations were 875 CTGs and 1950 CTG, while the ePAL in the immortalized cells was 654 CTGs, and the two main populations had a size of 953 CTGs and 2080 CTGs. Primary ADE-DM1 cells had an ePAL of 578 CTGs, and the highest expansion sizes of the most abundant populations (1505 CTG and 3075 CTG), while the immortalized cells, had an ePAL of 700 CTGs, and the two most abundant populations had a size of 1224 CTGs and 2301 CTGs. When analysing the expansion instability, GPM-DM1 had the lowest levels both in the primary and the immortalized cell lines, followed by JCC-DM1 and then ADE-DM1 (Figure 1B). All immortalized cell lines had some increase in expansion instability, which was between 6 and 25% of that observed in the parental cell lines, but the expansion instability pattern closely resembled the parental after the immortalization process. Interestingly, the larger the size of the most abundant CTG expansion and the greater degree of instability, the more severely affected the patient was. However, maybe due to the small sample size, no significant correlation was found.

### CTCF1 methylation level is increased in the myotonic dystrophy type 1 immortalized cell lines when compared with immortalized control cell lines as observed in primary cell lines

**Figure 2:**
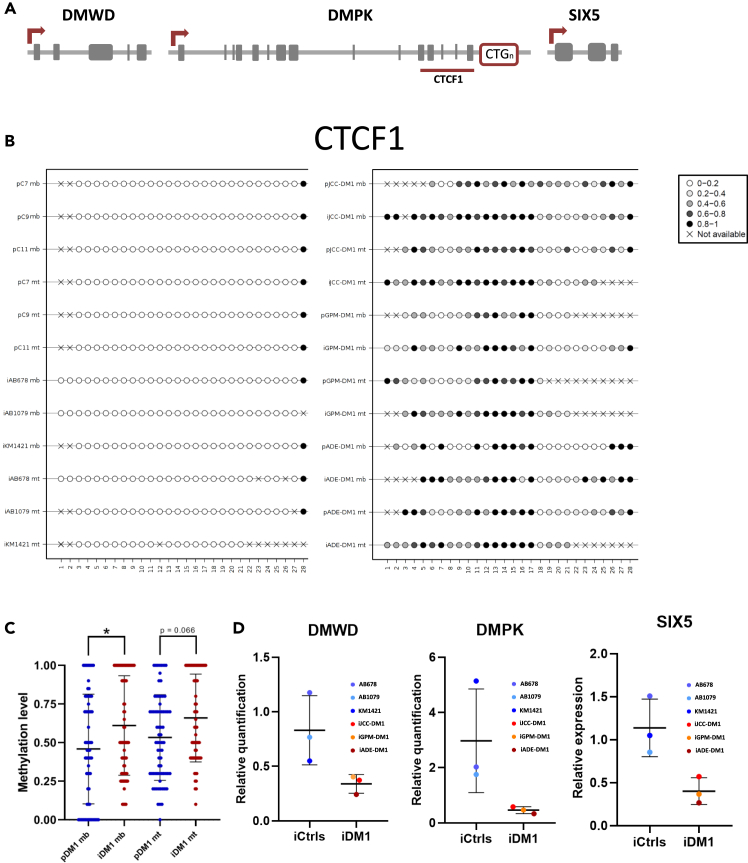
DNA methylation levels in CTCF1 is increased in immortalized cell lines when compared to primary cell lines (A) Schematic representation of the genomic *DMPK* locus. (B) Methylation plotter showing the methylation status of the CTCF1 region. Each circle represents a CpG dinucleotide. The level of methylation is represented by the gray gradient. (C) Graphical representation of the methylation levels in DM1 immortalized and primary myoblasts and myotubes. (D) Relative expression of *DMWD*, *DMPK* and *SIX5* genes at 5 days of differentiation. HPRT was used as housekeeping gene to normalize the data. All data are expressed as mean ± SEM. “p” and “i” before cell line name mean primary and immortalized, respectively. “mb” refers to myoblasts and “mt” to myotubes. Means were compared using unpaired two-tail Mann-Whitney test. ∗*p* ≤ 0.05

We analyzed DNA methylation levels of the CTCF1 region, which is located upstream of the CTG expansion in the *DMPK* gene (Figure 2A), in myoblasts and in 5 days differentiated myotubes derived from the primary and immortalized cell lines, in both controls and patients with DM1. There were significant differences in the level of methylation between controls and patients; while controls did not show methylation in the analyzed CpG sites of the CTCF1 region, patients with DM1 showed increased levels of methylation, both in myoblasts and in myotubes, in most of the CpG sites (Figure 2B). Moreover, we observed a significant increase in the methylation level in immortalized myoblasts compared to primary ones (Figure 2C). In the case of myotubes, a tendency (*p* = 0.06) was observed. No consistent differences were detected between patients regarding the methylation level of the CTCF1 site (Figure S1). To determine whether the methylation status of this region could change the expression level of neighboring genes, we analyzed the expression of *DMWD*, *DMPK*, and *SIX5* in immortalized myotubes from controls (iCtrls) and patients with DM1 (iDM1). Although significance was not reached due to dispersion in control cell values, all patient cell values were below any of the control values for the three genes (Figure 2D). More data would be needed to confirm the downregulation of DMWD, DMPK and SIX5, which could be a consequence of the hypermethylation in the CTCF1 region in DM1 muscle cells compared to controls.

### Myotonic dystrophy type 1 myoblasts show higher cell proliferation and reduction of early myogenic markers

**Figure 3:**
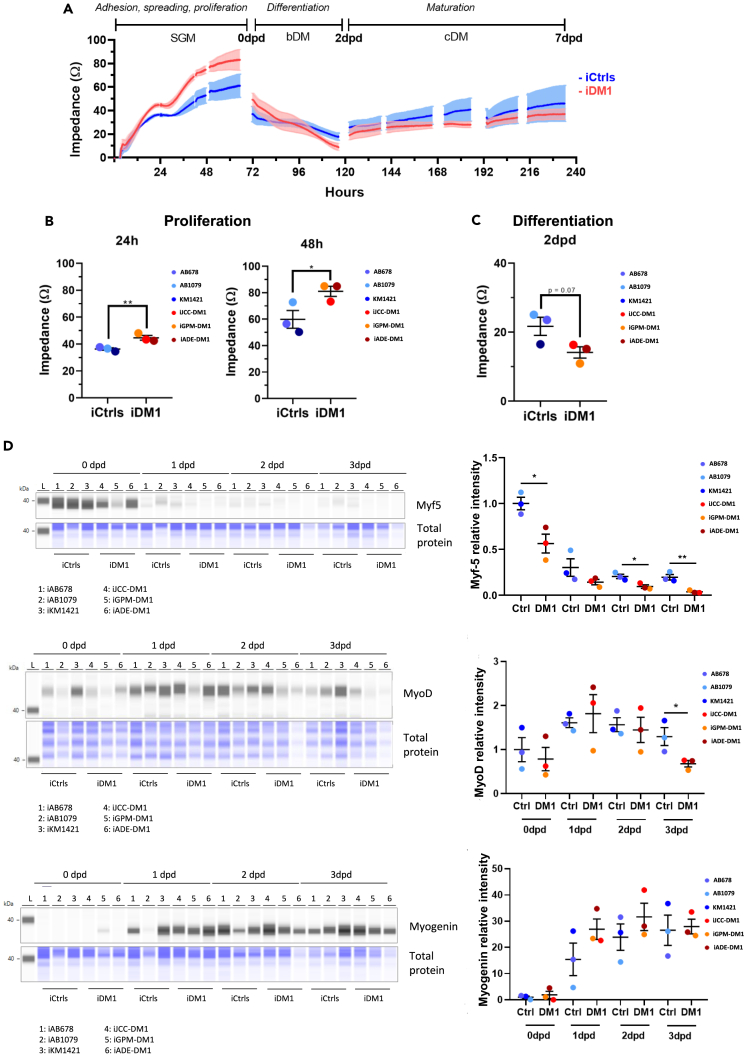
DM1 myoblasts show higher cell proliferation and reduced levels of early myogenic markers (A) Real-time impedance curves of human iCtrl (blue) and iDM1 (red) myoblasts during culture in proliferation medium (SGM) and differentiation media (bDM and cDM). (B) Proliferation of iCtrl (blue) and iDM1 (red) myoblasts was analyzed at 24 and 48 h after seeding. (C) Differentiation of iCtrl and iDM1 myoblasts was analyzed after 2 days in differentiation medium bDM (2dpd). (D) Jess Western blot analysis of Myf5, MyoD and Myogenin in iCtrl and iDM1 3 differentiating myoblasts at 4 different time-points: 0, 1, 2 and 3 dpd. Values are represented over Ctrl 0 dpd. Data information: *n* = 3 for iCtrl and iDM1. Dpd, days post differentiation. All data are expressed as mean ± SEM. (A–C) Dots indicate mean values of individual samples from 10 replicates. Means were compared using unpaired two-tail Student’s t test. ∗*p* ≤ 0.05, ∗∗*p* ≤ 0.01. “i” mean immortalized cell line.

We next sought to characterize the myogenic process in immortalized DM1 myoblasts by using real-time impedance analysis. Figure 3A shows the average real-time impedance curves of control (blue) and DM1 (red) myoblast samples throughout the myogenic process. Initially, all cultures increased their impedance values, which was indicative of myoblast adhesion, spreading, and proliferation, until they reached confluence, which resulted in achieving their maximum impedance values. Notably, at this step DM1 myoblasts showed a significant increase in impedance at 24 and 48 h after seeding, which was indicative of a higher adhesion, spreading, and proliferation levels in comparison to the control cells (Figure 3B). After switching to differentiation medium, a decrease in resistance was observed in both, control and DM1 cells, at 2 days post differentiation (dpd), due to cell reorganisation prior to muscle differentiation/fusion, with this decrease being higher in DM1 cells (Figure 3C). To address whether myogenic process could be altered in DM1 cells, we analyzed the levels of the myogenic regulatory factors Myf5, MyoD and myogenin, in time course experiments by Jess Western blots. Myf5 is expressed during stem cell activation and myoblasts proliferation; MyoD is expressed during myoblast proliferation, commitment to differentiation and myotube fussion; and myogenin is expressed during myotube fussion and maturation into myofiber.^47^ As shown in Figure 3D, Myf5 levels were significantly reduced in DM1 cells at 0,2,3 dpd, meanwhile MyoD was also significantly reduced at 3 dpd. No differences were found for Myogenin levels. In conclusion, DM1 cells show alterations in early myogenic markers My5 and MyoD.

### Myotonic dystrophy type 1 immortalized cells maintain a reduced fusion index as observed in their parental lines

**Figure 4:**
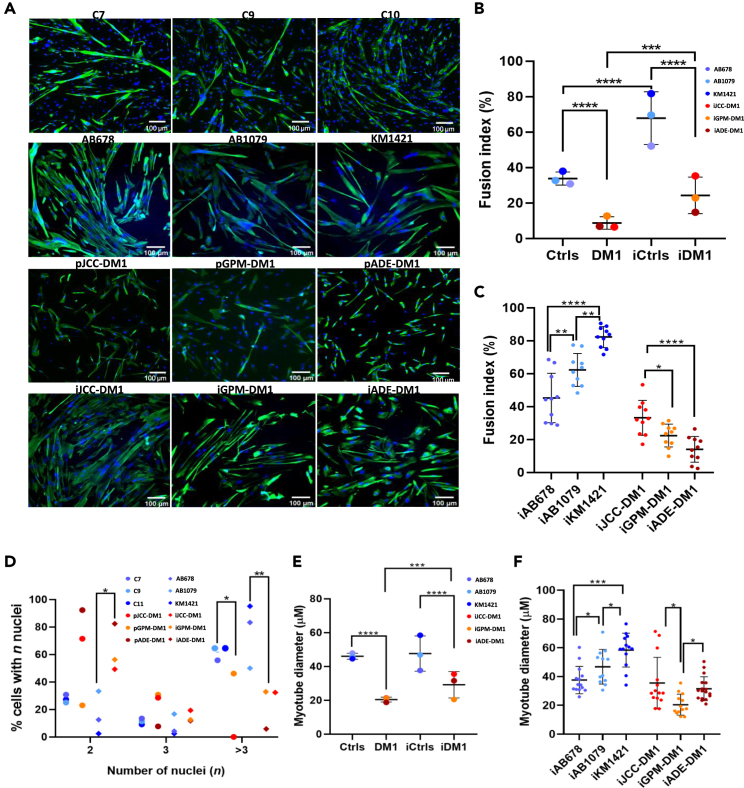
DM1 primary and immortalized myotubes present a lower fusion index compared to controls (A) Desmin (green) and nuclei (blue) immunofluorescence analysis performed in 5 days differentiated primary and immortalized DM1 or control myotubes. (B) Fusion index (% of nuclei in desmin positive myotubes with 2 or more nuclei) in primary and immortalized 5 days differentiated DM1 or control myotubes. (C and D) Fusion index (% of nuclei in desmin positive myotubes with 2 or more nuclei) in individualized immortalized 5 days differentiated DM1 or control myotubes D. Percentage of 5 days differentiated primary and immortalized myotubes containing 2, 3, >3 nuclei. (E) Myotube diameter (calculated using the maximum diameter value of desmin positive myotubes with 2 or more nuclei) in primary and immortalized 5 days differentiated DM1 or control myotubes. (F) Myotube diameter in individualized immortalized 5 days differentiated DM1 or control myotubes. All data are expressed as mean ± SEM. Means were compared using unpaired two-tail t-test. ∗*p* ≤ 0.05, ∗∗*p* ≤ 0.01, ∗∗∗*p* ≤ 0.001, ∗∗∗∗*p* ≤ 0.0001. “p” and “i” before cell line name mean primary and immortalized, respectively. In (B and C), dots indicate mean values of 5 individual analyzed images. In (B and D) at least 350 nuclei/cell line were analyzed. In (C) at least 850 nuclei/cell line were analyzed. In (E and F) at least 20 myotubes/cell line were analyzed.

Next, we investigated the fusion capacity of immortalized DM1 myotubes at 5 days of differentiation by desmin staining (Figure 4A). As shown in Figure 4B, the fusion index was significantly reduced in both primary and immortalized DM1 myotubes compared to the corresponding controls. We also observed that immortalized cells, both control and DM1, showed a higher fusion index than the parental cell lines (Figure 4B). When studied individually, we found significant differences in the fusion index between the three immortalized control cell lines and the three immortalized DM1 cell lines (Figure 4C). Moreover, we found differences in nuclei distribution in the myotubes (Figure 4D). We found a significant difference between patients and controls in the number of myotubes with more than three nuclei. Immortalized patients myotubes with two nuclei were more abundant than immortalized controls with two nuclei, reaching statistical significance. The number of myotubes with more than three nuclei was significantly higher in controls than in patients both in primary and immortalized cell lines (Figure 4D). Furthermore, the diameter of DM1 myotubes was dramatically reduced compared to the control ones (Figure 4E). When analysing individually the cell lines, differences in myotube diameter were found in all cell lines (Figure 4F). iGPM-DM1 was the cell line that had the smallest myotube diameter size when compared to the other cell lines. Altogether, the results demonstrate that the immortalized DM1 cell lines also show reduced muscle fusion capacity.

### Myotonic dystrophy type 1 immortalized myotubes maintain patient-derived heterogeneity and have equal or greater RNA foci and muscleblind-like 1 co-localization than their primary myotubes

**Figure 5:**
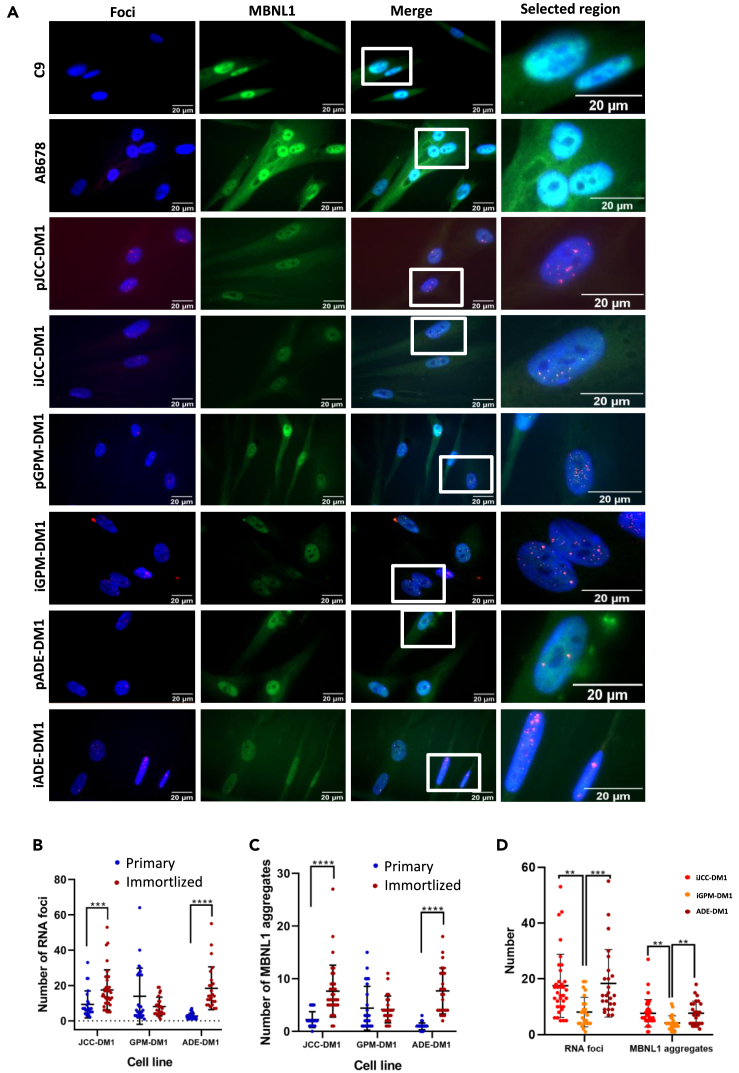
DM1 immortalized myotubes present equal or higher amount of RNA foci and MBNL1 aggregates than the original primary culture (A) Foci (red), MBNL1 (green) and nuclei (blue) immunofluorescence analysis performed in 5 days differentiated primary and immortalized DM1 myotubes. (B) Number of RNA foci/nucleus in primary and immortalized 5 days differentiated DM1 myotubes. (C) MBNL1 aggregates/nucleus in primary and immortalized 5 days differentiated DM1 myotubes. (D) Comparison of the number of RNA foci/nucleus and MBNL1 aggregates/nucleus between the three immortalized DM1 cell lines. All data are expressed as mean ± SEM. Between 25 and 35 DM1 nuclei and between 20 and 25 control nuclei were analyzed per cell line. “p” and “i” before cell line name mean primary and immortalized, respectively. Means were compared using unpaired two-tail Mann-Whitney test. ∗∗*p* ≤ 0.01, ∗∗∗*p* ≤ 0.001, ∗∗∗∗*p* ≤ 0.0001.

First, we checked for the presence of nuclear RNA foci in 5 days differentiated myotubes. Primary and immortalized control cells did not show RNA foci as expected (Figure S1), whereas primary DM1 myotubes showed variable numbers in the percentage of cells carrying RNA foci and in the number of RNA foci per cell (Figures 5A and 5B). Notably, this patient-derived heterogeneity with respect to the accumulation of RNA foci was greatest in immortalized DM1 cells. Interestingly, the JCC-DM1 and ADE-DM1 immortalized myotubes, which are the cells carrying the longest CTG expansions and derived from the most affected patients, showed not only a significantly higher number of RNA foci per cell compared to GPM-ADE (Figure 5D), but also the highest proportion of cells carrying more than 10 foci per cell (Table 3).

**Table 3:** Detailed data of the RNA foci and MBNL1 aggregates found in 5 days differentiated primary and immortalized myotubes

Cell line	Foci avg.	% of cells with 0 foci	% of cells with 1–5 foci	% of cells with 6–10 foci	% of cells with 11–15 foci	% of cells with 16–20 foci	% of cells with >20 foci	MBNL1 avg.	% of cells with 0 MBNL1 aggr.	% of cells with 1–5 MBNL1 aggr.	% of cells with 6–10 MBNL1 aggr.	% of cells with 11–15 MBNL1 aggr.	% of cells with 16–20 MBNL1 aggr.	% of cells with >20 MBNL1 aggr.
pJCC-DM1	9,28	0	32	44	8	4	12	2,24	4	96	0	0	0	0
iJCC-DM1	17,51	0	8,6	20	22,9	25,7	22,9	7,63	0	34,3	45,7	14,3	2,85	2,85
pGPM-DM1	13,88	0	48	20	0	0	32	4,4	0	72	20	8	0	0
iGPM-DM1	8,08	0	44	28	12	16	0	4,16	0	76	20	4	0	0
pADE-DM1	2,69	0	91,4	8,6	0	0	0	0,94	20	80	0	0	0	0
iADE-DM1	18,40	0	0	24	36	12	28	7,68	0	40	28	28	4	0

“p” and “i” before cell line name mean primary and immortalized, respectively.

It is well known that staining with an anti-MBNL1 antibody shows the presence of MBNL-1 aggregates, which co-localize with RNA foci only in DM1 cells. This aggregation of MBNL1 leads to a decrease in cytoplasmic and nuclear fluorescence of MBNL1 compared to that of control cells (Figure 5A). As we observed with RNA foci, primary DM1 cells had a variable number of co-localized aggregates. Immortalized JCC-DM1 and ADE-DM1 myotubes showed a significantly higher mean number of MBNL1 aggregates, as well as a high percentage of cells with more than 10 MBNL1 aggregates when compared to primary cell lines (Figure 5D; Table 3). Altogether, these results indicate that these DM1 immortalized cell lines also show the cell heterogeneity associated with DM1 tissues.

### Myotonic dystrophy type 1 immortalized myotubes showed the same pathological splicing alterations than their primary myotubes

**Figure 6:**
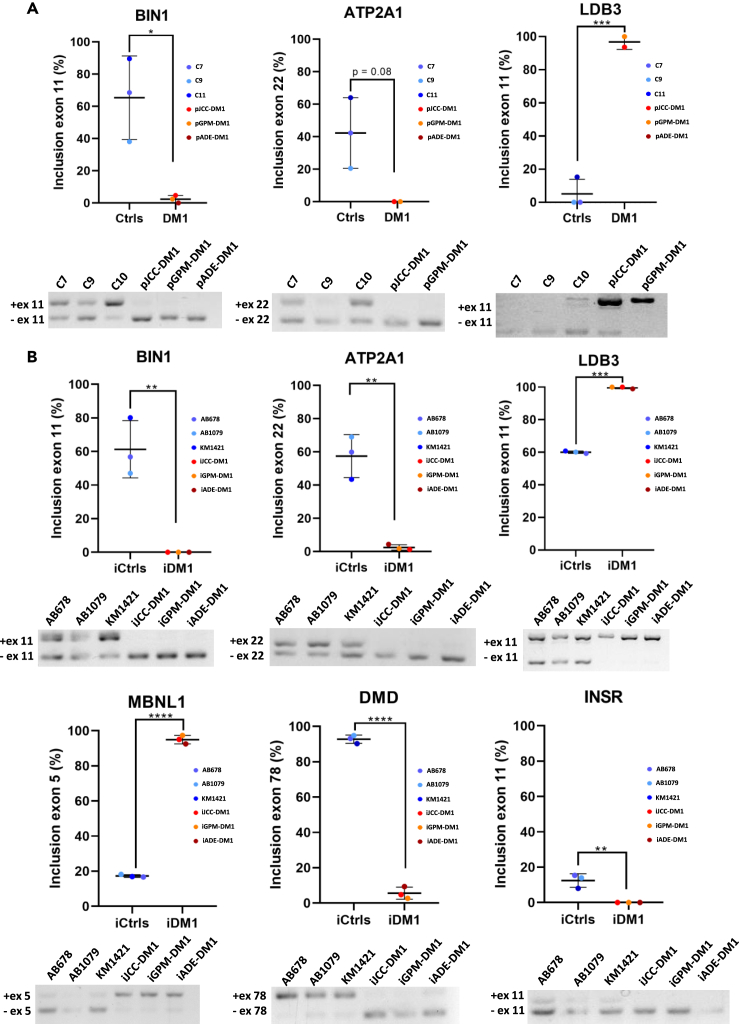
Immortalized DM1 myotubes maintain the splicing defects that characterize DM1 primary myotubes (A) Exon inclusion analysis of *BIN1, LDB3* and *ATP2A1* in primary control and DM1 5 days differentiated myotubes. (B) Exon inclusion analysis of *BIN1, MBNL1, LDB3, INSR, DMD* and *ATP2A1* in immortalized control and DM1 5 days differentiated myotubes. All data are expressed as mean ± SEM. 3 DM1 samples and 3 control samples were analyzed in each splicing both in primary and immortalized samples, except for *ATP2A1* and *LDB3* in primary samples where 2 DM1 samples and 3 controls were analyzed. Means were compared using unpaired two-tail t-test. ∗*p* ≤ 0.05, ∗∗*p* ≤ 0.01, ∗∗∗*p* ≤ 0.001, ∗∗∗∗*p* ≤ 0.0001. “p” and “i” before cell line name mean primary and immortalized, respectively.

**Figure 7:**
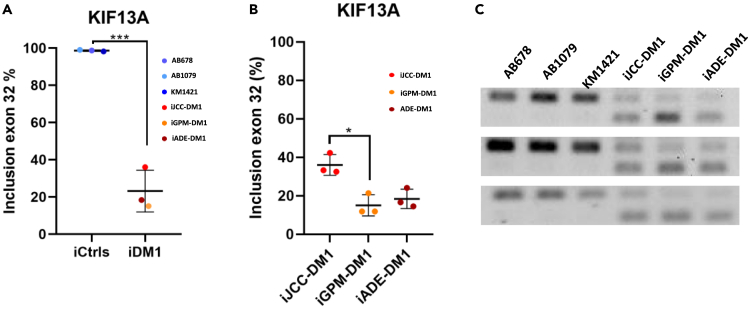
KIF13A splicing defect is heterogeneously expressed among immortalized cell lines (A) Exon inclusion analysis of KIF13A in immortalized control and DM1 5 days differentiated myotubes. (B) Exon inclusion analysis of KIF13A in immortalized 5 days differentiated myotubes derived from the three DM1 cell lines. (C) Gel analysis of *KIF13A* splicing analysis in control and patient DM1 myotubes. All data are expressed as mean ± SEM. *n* = 3 for each cell line. Means in (A) were compared using unpaired two-tail t-test and in (B) with ANOVA. ∗*p* ≤ 0.05, ∗∗∗*p* ≤ 0.001. “i” before cell line name means immortalized.

Next, we analyzed splicing alterations in immortalized DM1 myotubes. We addressed the splicing pattern of transcripts previously described as altered in patients with DM1 (*BIN1* (exon 11), *MBNL1* (exon 5), *LDB3* (exon 11), *INSR* (exon 11), *DMD* (exon 78), and *ATP2A1* (exon 22)). As shown in Figure 6A, DM1 primary myotubes have significantly different *BIN1* and *LDB3* splicing patterns, while a *p*-value of 0.08 was observed in *ATP2A1* splicing. The same results were obtained when analysing immortalized DM1 myotubes and comparing them with control cells (Figure 6B). Moreover, we found a splicing alteration in the KIF13A gene that was heterogeneously expressed among DM1 immortalized myotubes (Figure 7A). IJCC-DM1 myotubes had a significantly higher exon 32 inclusion than iGPM-DM1 myotubes (Figure 7B), as it can be observed in the agarose gels (Figure 7C). These results indicate that immortalized DM1 myotubes maintain the splicing defects that characterise this disease, although they can show heterogeneity in the levels of splicing alteration.

### Antisense oligonucleotide treatment is equally efficient in myotonic dystrophy type 1 immortalized cell lines as in their original parental lines

**Figure 8:**
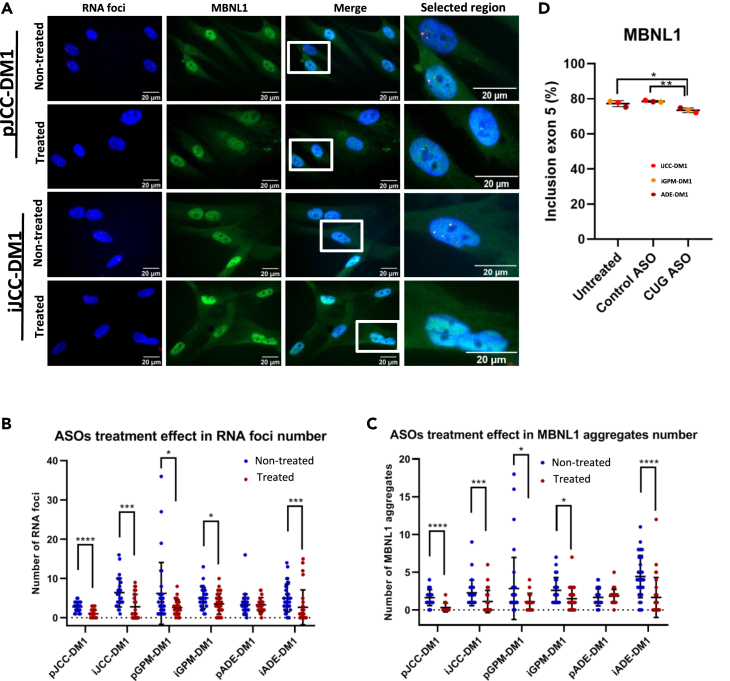
Immortalized DM1 myotubes respond to treatment in a similar way to primary DM1 myotubes (A) Foci (red), MBNL1 (green) and nuclei (blue) immunofluorescence analysis performed in 9 days differentiated primary and immortalized DM1 myotubes. Rows 1 and 3 correspond to non-treated cells while rows 2 and 4 correspond to 48 h ASO-treated cells. (B) Number of RNA foci/nucleus in primary and immortalized 48 h ASO-treated or non-treated 9 days differentiated DM1 myotubes. (C) Number of MBNL1 aggregates/nucleus in primary and immortalized 48 h ASO-treated or non-treated 9 days differentiated DM1 myotubes. (D) MBNL1 splicing analysis in immortalized 48 h ASO-treated, control-treated or non-treated 9 days differentiated myotubes. All data are expressed as mean ± SEM. For each cell line in (B and C), it was analyzed between 29 and 43 DM1 nuclei. In (D), 3 DM1 samples were analyzed for each condition. “p” and “i” before cell line name mean primary and immortalized, respectively. Means were compared using unpaired two-tail Mann-Whitney test. ∗*p* ≤ 0.05, ∗∗∗*p* ≤ 0.001, ∗∗∗∗*p* ≤ 0.0001.

To test whether these immortalized cell lines would be suitable for testing DM1 treatments, we treated the cells with an ASO targeting the CTG expansion,^48^ previously used in our laboratory (unpublished data). We measured the effect of treatment by analysing the number of RNA foci and colocalizing aggregates of MBNL1 (Figures 8A and S3). Importantly, we observed a significant reduction in both the number of RNA foci (Figure 8B) and the MBNL-1 colocalizing aggregates (Figure 8C) in all immortalized DM1 cell lines. These reductions were also observed in two of the primary cell lines, JCC-DM1 and GPM-DM1, but not in ADE-DM1. Notably, the MBNL-1 colocalizing aggregates decrease was associated in some cases with an increase in the fluorescence signal of MBNL1 in the nucleus. To test whether the RNA foci reduction caused splicing restoration we studied the effect of the ASO treatment in the usual DM1 splicing alterations. Notably, we found a significant reduction in MBNL1 exon 5 splicing alteration. (Figure 8D).

## Discussion

Heterogenicity in molecular, clinical, and functional parameters in DM1 is often not represented in the cellular models used to study this disease. The need to generate cellular models that mimic the reported patient differences is increasing, especially with the current development of therapies, and with the necessities to understand pathophysiological mechanisms of DM1. To have more robust results, it is important to work with cell cultures derived from DM1 muscle biopsies, which can show patient heterogeneity and preserve their natural genomic context. However, the accessibility and availability of muscle biopsies from patients with DM1 is very limited, and primary muscle cultures show a reduced proliferative capacity after few passages. In this study, we have generated three immortalized human muscle cell lines derived from three different subtypes of patients with DM1 (juvenile, adult, and late-onset). The genetic, epigenetic, and molecular characterization of these cellular models have shown that all three present the hallmarks of the disease and importantly, they are heterogeneous both from a clinical and molecular point of view.

Given the high instability of the CTG repeat in DM1, we performed SP-PCR to better characterize the genetic expansion variability in our cellular models. Our results revealed high heterogeneity in CTG expansion, with two of the patient-derived cells (ADE-DM1 and JCC-DM1) showing higher ePAL (above 500) and higher instability (above 1900) than the other cell line (GPM-DM1, below 500 and 700, respectively). In addition, the patient-derived muscle cell line with the highest instability (ADE-DM1) was the one whose most abundant populations had the largest CTG expansions and interestingly, corresponded with the most severely affected patient, both from the cardiac and functional points of view. In contrast, the patients with less instability (JCC-DM1 and GPM-DM1) were the mildest affected patients, both cardiacally and functionally. Somatic instability of the CTG expansion has been previously described to be a contributor of disease severity,^49^ yet the major contributor of the severity is the ePAL. In this case, the highest ePAL was also found in the immortalized cells of the patient with the highest disease severity (ADE-DM1). However, the primary cells derived from this same patient did not have the highest ePAL, in fact JCC-DM1 had the highest one of the primary cells. These differences between primary and immortalized cells must be derived from the clonal selection that takes place during the immortalization process, in which the CTG expansion tends to expand, but can also contract.

It is indeed remarkable, that clonal cells originating from a single CTG expansion end up having a very similar instability to the primary cells they derived from. It has been hypothesized that individual specific differences, as well as environmental or genetic factors, may contribute to somatic instability.^49^ Although 40% of the variance has been reported to be attributed to genetic factors, our results indicate that the contribution of the genetic factors should be above 75% (since only between 6 and 25% variation in instability was found between parental and immortalized cell models). In addition, it is likely that part of the instability was not detected in our study, since measuring these CTG expansions was challenging, and muscle, unlike other tissue such as blood, has larger CTG expansions that are difficult to detect by SP-PCR.^50^^,^^51^^,^^52^ Deep genomic sequencing techniques^53^^,^^54^ would probably give more accurate and precise information regarding the larger CTG fragments in muscle and the instability of CTG expansion in DM1 samples.

The CTG expansion overlaps with a 3.5 kb CpG island flanked by two CTCF binding sites, named CTCF1 and CTCF2. We and others have reported changes in the DNA methylation pattern of the CTCF1 region in blood samples from DM1.^10^^,^^11^^,^^12^ In addition, our group also demonstrated that the CTCF1 region was methylated in a tissue-specific manner only in DM1 muscle biopsies (and not in skin or blood from the same patients), as well as in primary DM1 muscle cells, whereas tissues from unaffected individuals were completely unmethylated.^12^ Importantly, the DM1 immortalized muscle cells showed the previously reported CTCF1 hypermethylation in both myoblasts (muscle progenitor cells) and myotubes (mature muscle cells), indicating that the DNA methylation alterations were conserved. However, we observed a significant difference in the methylation levels between primary and immortalized cell lines. Immortalized cell line methylation levels were increased compared to the primary ones. This can be explained by the observation that cellular models, especially immortalized cell lines that have been in culture for a substantial amount of time (since they are originated from a single cellular clone), can increase DNA methylation levels^55^^,^^56^ and/or because of the purity of cell cultures. The presence of a small portion of fibroblasts in the primary cultures, which have an unmethylated CTCF1 region, can have an impact in these CTCF1 methylation studies. DNA methylation is considered a repressive epigenetic mark that plays a role in gene silencing.^57^^,^^58^^,^^59^ Although we did not find a significant reduction of DMPK, SIX5, and DMWD in our study, probably due to a small sample, it has been previously reported that there has been a decrease in the expression of these genes in DM1.^60^^,^^61^^,^^62^ This reduction could be suggesting that DNA hypermethylation in the CTCF1 region could alter transcriptional activity in DM1 muscle samples, which could affect muscle functions. Notably, *SIX5* is abundantly expressed in muscle, heart, brain, and eyes, which are tissues that are affected in DM1. It is homologous to the Drosophila eye development gene sine oculis and it has been proven that *SIX5* deficient mice develop cataracts, as observed in patients with DM1.^63^ The *DMWD* gene is abundantly expressed in the brain and testis,^64^ although its function is still not clear. However, very recently a quadruple mutant mouse was generated in which the expression of *SIX5, DMWD, DMPK,* and *MBNL1* was reduced, and these mice recapitulate many important manifestations in congenital DM1, suggesting that changes in gene expression in genes at the *DMPK* locus may modulate disease severity.^65^

The immortalized DM1 cellular models showed an increased proliferation rate when compared with immortalized control models. An advantage in growth and proliferation has already been described in immortalized DM1 lymphocyte cell lines.^66^ This effect has been termed “mitotic drive” and it explains why the CTG region tends to expand. Khajavi and colleagues demonstrated that immortalized lymphocytes carrying longer CTG expansions had a growing advantage due to increased Ras, Erk1, and Erk2 activity and decreased p21^WAFI^ activity. However, once DM1 immortalized muscle cell models stop growing and are stimulated to differentiate, they start having alterations.^44^ In our immortalized cellular models, DM1 immortalized cells showed a decrease in the impedance during the myotube fusion process, reduced levels of early myogenic markers, a diminished fusion capacity with smaller myotubes, and lower nuclei number per myotube. Some of these alterations have also been found in primary muscle derived cells and have been linked to abnormalities in the temporal expression of differentiation regulators, myogenic progression markers, and alternative splicing patterns before and immediately after the onset of differentiation.^67^ These alterations are intrinsically linked to the CTG expansion, since the excision of the expanded repeat reverts all these abnormalities.^67^ However, some influence may also come from the environment, since studies in 3D models with immortalized cells demonstrated that these differentiation deficiencies were attenuated^68^ when a better niche for differentiation was utilized. Moreover, when comparing primary and immortalized myotubes, we observed an increase in the fusion index in immortalized cell lines, both in controls and DM1. As discussed with the methylation pattern in CTCF1, it is likely that this difference is due to the greater purity of immortalized cultures compared to primary ones. Regarding heterogeneity in the fusion index between cell lines, we found differences between both immortalized controls and patients; meaning that other factors may contribute to the fusion capacity.

Analysis of the molecular hallmarks of the disease revealed heterogeneous alterations in the accumulation of RNA foci and in the sequestration of MBNL1 in our immortalized cell models. Importantly, we have observed that the higher the CTG instability, the higher the average RNA foci per cell. ADE-DM1, had an instability of 3068 CTGs, and had a mean of 18,40 foci per nucleus; JCC-DM1 had an instability of 2467 CTGs and a mean of 17,51 foci per nucleus, and GPM-DM1 had an instability of 828 CTGs and an average of 8,08 foci per nucleus. A direct association between CTG size and foci number in muscle cells has been reported before by our group.^23^ We hypothesize that although the CTG size would determine the number of RNA foci at the single cell level, the instability of the CTG repeat in the cells of a certain tissue, would determine the mean of the RNA foci in this particular tissue.^18^^,^^24^^,^^30^

MBNL1 sequestration was also dependant on the RNA foci average. So, the higher RNA foci/cell, the more MBNL1 aggregates/cell colocalizing with those foci. Again, ADE-DM1 is the cell line carrying more MBNL1 aggregates (7,68 per myotube), followed by JCC-DM1 (7,63 aggregates per myotube), and finally, GPM-DM1 (carrying 4,16 aggregates per myotube). So, it is clear that there is a relationship between CTG expansion, *DMPK* pathological transcript accumulation, and MBNL1 trapping. Heterogeneity in RNA foci accumulation and MBNL1 sequestration can be modified by different experimental conditions. A lower number of RNA foci was observed in the immortalized patient cell lines with 9 differentiation days, when compared to 5 differentiation days. We hypothesize that the heterogeneity could be due to the higher mortality rate observed after 9 days of differentiation, which may difficult the detection of RNA foci.

Splicing alterations were also found in our cellular models. We observed alterations in the splicing of *ATP2A1* and *SERCA1*, which is observed only in mature skeletal muscle cells and is sometimes hard to detect in patient primary cultures^34^^,^^69^ and in patient derived iPSCs that are differentiated to myotubes.^70^ Our cell models showed alterations in *BIN1, ATP2A1, MBNL1, LDB3, INSR*, *DMD* and *KIF13A*, which have previously been found altered in DM1 muscle cell models.^44^ We found heterogenicity in the alterations of *KIF13A* with JCC-DM1 having significantly less alterations than GPM-DM1. *KIF13* codes for a gene involved in the positioning of endosomes.^71^ It is likely that heterogenicity is present in other splicing alterations, but more global transcriptomic studies would be needed to further determine this heterogenicity.

The generation of specific therapies^72^ targeting either the gene through CRISPR-Cas9,^73^^,^^74^^,^^75^ the foci,^76^^,^^77^ or the increase in MBNL1 availability^78^^,^^79^ would make these cells an attractive cellular model in which the heterogeneous defects are present in different proportions, which can be quantified after treatments. Notably, the three models showed a significant reduction of RNA foci and MBNL1 aggregates after being treated with an ASO directed against the CTG expansion.^48^ Moreover, a significant recovery of the *MBNL1* splicing alteration was observed. Overall, these models would allow treatments to be tested on a larger and more heterogeneous sample than was previously possible.

Prior to this publication, only four DM1 immortalized muscle cellular models have been developed by different research groups (Table 4).^44^^,^^45^ These cellular models have been a powerful tool used to discover knowledge, develop therapeutic strategies, and overcome the challenges presented by artificial, primary, or transdifferentiated disease models. However, heterogenicity in molecular, clinical, and functional parameters was not represented even though it is a hallmark of the disease.

**Table 4:** Comparative table of the characterization of the immortalized DM1 myoblasts/myotubes models available

Cell line	Age of onset/Clinical subtype	Disease progression at sampling	CTG expansion	Epigenetics	RNA foci number	MBNL1 aggregates	Splicing alterations	Differentiation studies	Treatment assessment	Publication
**JCC-DM1**	15	✓	✓	✓	✓	✓	✓	✓	✓	Núñez-Manchón et al. 2023
**GPM-DM1**	42	✓	✓	✓	✓	✓	✓	✓	✓	Núñez-Manchón et al. 2023
**ADE-DM1**	27	✓	✓	✓	✓	✓	✓	✓	✓	Núñez-Manchón et al. 2023
**DM1**	Infantile	NA	✓	NA	✓	✓	✓	✓	✓	Arandel et al. 2017^44^
**DM#1**	NA	NA	✓	NA	✓	NA	✓	✓	✓	Pantic et al. 2016^45^
**DM#2**	NA	NA	✓	NA	✓	NA	✓	✓	✓	Pantic et al. 2016^45^
**DM1**	Congenital	NA	✓	NA	NA	NA	NA	NA	NA	Bigot et al. 2009^46^

NA, not available.

In this article, we have presented three immortalized muscle cell models for the study of DM1. We have demonstrated that these immortalized cell models behave similarly to the parental cells from which they derive. However, they are heterogeneous, and harbor different molecular alterations that are linked to the presentation of different clinical symptoms in patients. These models will offer new possibilities to understand DM1 from a more diverse point of view. The cells will serve as a tool to study and quantify the degree of certain molecular alterations, and to assess the efficacy of different therapies in a context of different molecular alterations in different genetic backgrounds. Overall, we have generated three cellular models for DM1 that we expect will contribute to the better understanding of this pathology from a more diverse point of view.

### Limitations of the study

The main limitation of this study was the limited amount of immortalized cell lines that we were able to generate as it makes it challenging to obtain significant statistical results. Furthermore, these cells were only obtained from females, so there was a sex bias in the data obtained from these three immortalized models.

Another limitation of the study was the unfeasibility of working with the same passage for all the analyzed DM1 cells, which might affect the outcome of some experiments. We observed that the primary cell line with 7 passages, ADE-DM1 was the one that had significantly less RNA foci accumulation and less MBNL1 aggregates than any other (Figure 5). In addition, this cell line was also not responding to the treatment (Figures 8 and S3). Previous unpublished data from our lab showed that this patient-derived primary cell line responded well to this same treatment but at earlier passages. Working with passage 7 may involve cellular alterations, like senescence, which could affect transcriptional activity and response to antisense oligonucleotide treatment.

## STAR★Methods

### Key resources table

**Table:** 

REAGENT or RESOURCE	SOURCE	IDENTIFIER
**Antibodies**		

Mouse anti-MBNL1	DSHB	Cat# MB1a(4A8), RRID:AB_2618248)
Mouse monocolonal anti-Desmin	Abcam	Cat# ab8470, RRID:AB_306577
Rabbit polyclonal MyoD Antibody	Santa Cruz Biotechnology	Cat# sc-304, RRID:AB_631992
Mouse monocolonal anti- myogenin	Santa Cruz Biotechnology	Cat# sc-12732, RRID:AB_627980
Rabbit polyclonal Myf-5 antibody	Santa Cruz Biotechnology	Cat# sc-302, RRID:AB_631994

**Bacterial and virus strains**		

hTERT lentiviral vectors	Mamchaoui et al.^83^	NA
Cdk4 lentiviral vectors	Mamchaoui et al.^83^	NA

**Critical commercial assays**		

EZ DNA Methylation Gold kit	Zymo Research	Cat#D5005

**Experimental models: Cell lines**		

Human primary DM1 myoblasts: JCC-DM1, GPM.DM1, ADE-DM1	This paper	NA
Human primary control myoblasts: C7, C9, C11	This paper	NA
Human immortalized DM1 myoblasts: iJCC-DM1, iGPM.DM1, iADE-DM1	This paper	NA
Human immortalized control myoblasts: AB678, AB1079, KM1421	This paper	NA

**Oligonucleotides**		

DIG-labelled LNA (CAG)_7_	MOLBIOL	NA
CTCF1 Fwd1 5′-TGTYGTYGTTTTGGGTTGTATTG-3′	ThermoFisher	NA
CTCF1 Rev1 5′-TTCCYGACTACAAAAACCCTTYG-3′	ThermoFisher	NA
CTCF1 Fwd2 5′-GTTGTATTGGGTTGGTGGTTTA-3′	ThermoFisher	NA
CTCF1 Rev2 5′-CTACAAAAACCCTTYGAACCC-3′	ThermoFisher	NA
Primers for qPCR, see Table S1	ThermoFisher	NA
Primers for splicing analysis, see Table S2	ThermoFisher	NA
BNANC gapmer with the sequence AGCagcagcagCAG	Bio-Synthesis	NA
101 Rev 5′-CTTCCCAGGCCTGCAGTTTGCCCATC-3′	ThermoFisher	NA
102 Fwd 5′-GAACGGGGCTCGAAGGGTCCTTGTAGC-3′	ThermoFisher	NA

**Software and algorithms**		

GelAnalyzer 19.1	GelAnalyzer	http://www.gelanalyzer.com/?i=1
Chromas version 2.6.6.	Technelysium	https://technelysium.com.au/wp/chromas/
LightCycler 480	Roche	https://diagnostics.roche.com/global/en/products/instruments/lightcycler-480-ins-445.html
AxIS Z	Axion Biosystems	https://www.axionbiosystems.com/resources/product-brochure/axis-z-21-cfr-part-11-statement
ZEN blue	ZEISS	https://www.zeiss.com/microscopy/es/productos/software/zeiss-zen.html
GraphPad Prism 8	Graphpad	https://www.graphpad.com/
ImageJ	NIH	https://imagej.nih.gov/ij/download.html

**Other**		

Methylation Plotter web tool	Mallona et al.^84^	http://maplab.imppc.org/methylation_plotter/

### Resource availability

#### Lead contact

Further information and requests for resources and reagents should be directed to and will be fulfilled by the lead contact, Gisela Nogales-Gadea (gnogales@igtp.cat).

#### Materials availability

Immortalized cell lines iJCC-DM1, iGPM-DM1 and iADE-DM1 are available upon reasonable request.

#### Data and code availability

• All data reported in this paper will be shared by the lead contact upon request.
• This paper does not report original code.
• Any additional information required to reanalyse the data reported in this paper is available from the lead contact upon request.

### Experimental model and study participant details

#### Sample collection and patient characterization

This study was approved by the Ethics Committee of the University Hospital Germans Trias i Pujol and was performed in accordance with the Declaration of Helsinki for Human Research. Written informed consent was obtained from all participants. Three genetically confirmed DM1 patients with different clinical features aged 36, 39 and 42 were selected to perform the immortalization of biopsy-derived primary myoblasts. The biopsies from the patients were obtained from left biceps. Two different types of controls, obtained from different individuals, were used: primary controls, whose biopsies were obtained from intrinsic hand muscles and immortalized controls that were obtained from healthy individuals quadriceps (AB678, AB1079) and paravertebral muscles (KM1412). Clinical information of DM1 patients was obtained from medical records.

#### Cell culture and immortalization

Primary myoblasts were isolated from muscle biopsy explants on culture plates treated with human plasma and gelatin 1.5% and then purified by CD56 magnetic separation according to manufacturer’s instructions (Miltenyi Biotec; Bergisch Gladbach). Primary myoblasts were grown on 0.1% gelatin-coated flasks in proliferation medium containing Dulbecco’s Modified Eagle’s Medium (DMEM) supplemented with 10% FBS, 22% M-199, PSF 1x, 10 μg/mL insulin, L-glutamine 2 mM, 25 ng/mL FGF and 5 ng/mL EGF. At 80–90% confluence proliferation medium was substituted by differentiation medium containing DMEM supplemented with 2% FBS, 22% M-199, PSF 1×, 10 μg/mL insulin and L-glutamine 2 mM. For immortalization, 50.000 primary myoblasts were transduced with hTERT and Cdk4 lentiviral vectors with a MOI of 3 in the presence of 4 μg/mL of polybrene (Sigma-Aldrich) overnight. 48 h after, transduced cell cultures were selected with puromycin (1 μg/mL, LifeTechnologies) for 6 days and neomycin (0.1 mg/mL, Life Technologies) for 10 days. Cells were then seeded at clonal density (2 cells per cm2) for 10 days. Selected individual myogenic clones were isolated from each population, using glass cylinders, and their proliferation and differentiation capacities were evaluated. We selected clones which were able to proliferate and to differentiate correctly (we tested their ability to differentiate into myotubes, using immunostaining with MF20 antibody, which recognizes all skeletal-muscle myosin heavy chains (MyHCs). We removed the non-myogenic clones. ^83^ Immortalized myoblasts were grown on uncoated flasks in proliferation medium containing DMEM supplemented with 16% M-199, 20% FBS, Gentamycin 50 μg/mL, fetuin 25 μg/mL, hEGF 5 ng/mL, bFGF 0.5 ng/mL, Insulin 5 μg/mL and dexamethasone 0,2 μg/mL. For differentiation experiments cells were grown in 1:100 matrigel matrix (Corning) coated surfaces until 80–90% confluence. Proliferation medium was substituted by differentiation medium containing DMEM supplemented with 10 μg/mL of insulin and 50 μg/mL Gentamycin. Both primary and immortalized myoblasts were differentiated into myotubes for 5 or 9 days, depending on the experiment. Pellets for RNA and DNA analysis were collected and coverslips for FISH and ICC were fixed with 4% PFA and permeabilized with 0.3% Triton. We performed an STR variant analysis of 16 locus for cell authentication purposes. Mycoplasma test was performed both before and after the immortalization and it turned out negative for all the cell lines.

### Method details

#### CTG expansion sizing

DNA from the primary and immortalized myotube cultures was extracted using the PureLink Genomic DNA Mini Kit (Invitrogen) according to the manufacturer’s instructions. To determine CTG expansion size we performed a small-pool PCR from EcoRI digested DNA followed by Southern blotting. The PCR was performed with 5 ng of digested DNA, using Expand Long Template PCR System (Roche) and primers 102 (5′-GAACGGGGCTCGAAGGGTCCTTGTAGC-3′) and 101 (5′-CTTCCCAGGCCTGCAGTTTGCCCATC-3′). The conditions of the PCR were divided in four steps: 1) 3′ at 96°C. 2) 30″ at 65°C followed by 3′ at 68°C and 30″ at 95°C for 10 cycles. 3) 30″ at 65°C followed by 3′ at 68°C, which increase the duration 30″ each cycle and 30″ at 95°C for 15 cycles. 4) 1′ at 65°C and 8′ at 68°C. PCR products were run in an agarose gel (Serva) ON at 4°C and transferred into a nylon membrane. The membrane is cross-linked and incubated with a DIG-labelled (CAG)_7_ LNA probe at 65°C for 2 h. The membrane is finally developed using anti-DIG alkaline phosphatase and CDP-star (Roche) according to the manufacturer’s instructions. The progenital and modal alleles of each culture were estimated through comparison against the molecular weight ladder GeneRuler 1Kb (ThermoScientific) using GelAnalyzer 19.1 software. The progenital allele length was estimated as the 10^th^ percentile of allele frequency distribution. The modal allele length was determined as the most frequent allele. The level of somatic instability was calculated by subtracting the 10^th^ percentile from the 90^th^ percentile.

#### DNA methylation analysis

DNA was bisulphite converted using the EZ DNA Methylation Gold kit (Zymo Research), according to the manufacturer’s protocol. Bisulphite-converted DNA was amplified by nested PCR for the CTCF1 region (located upstream of the CTG repeat in the *DMPK* gene) with the TaKaRa Taq DNA polymerase (TaKaRa Bio Inc.). For the first PCR, 50 ng of bisulphite-converted DNA were used, while for the second PCR 3 μL of the first PCR product were used. Primers sequences were the following: CTCF1 F1 5′-TGTYGTYGTTTTGGGTTGTATTG-3′, CTCF1 R1 5′-TTCCYGACTACAAAAACCCTTYG-3′, CTCF1 F2 5′-GTTGTATTGGGTTGGTGGTTTA-3′, CTCF1 R2 5′-CTACAAAAACCCTTYGAACCC-3'. PCR conditions for both amplifications were: 5 min of initial denaturation at 94°C, 40 cycles of 30 s denaturation at 94°C, 30 s annealing at 57°C, and 30 s of extension at 72°C and a final extension of 5 min at 72°C. Amplicons were purified using Illustra ExoProStar 1-Step (Merck), according to the manufacturer’s instructions. Purified products were sequenced using the BigDye Terminator v3.1 cycle sequencing kit (Thermo Fisher Scientific), following the manufacturer’s guidelines. Sequencing products were run on an ABI Prism 3130 Genetic Analyzer (Applied Biosystems) and were analyzed with Chromas software version 2.6.6. The data obtained was represented with the Methylation Plotter web tool.^84^

#### Expression analysis by qPCR

RNA was extracted from 5 days differentiated myotubes using PureLink RNA Mini Kit (Invitrogen) according to the manufacturer’s instructions. 500 ng of RNA was retrotranscribed using SuperScript IV reverse transcriptase (Thermo Fisher Scientific). cDNA was amplified by qPCR in a LightCycler 480 using the Lightcycler 480 SYBR Green I Master (Roche). The primers used are listed in Table S1. Amplification consisted of 40 cycles with the following conditions: 10 s at 95°C for denaturation, 10 s at 65°C for annealing and 15 s at 72°C for extension. Results were analyzed with the LightCycler 480 software.

#### Impedance measurements

Real-time impedance measurements were used for addressing myogenic behavior of control and DM1 immortalized human myoblasts, by using the Maestro Edge equipment with the impedance module (Axion BioSystems). Prior to cell seeding, Cytoview Z 96-well plates (Axion BioSystems) were overlaid with 100 μL of culture media and placed into the Maestro Edge to record baseline readings of the background impedance without cells. Afterward, myoblasts were seeded on the plates at 20,000 cells per well and left 1 h at room temperature to ensure homogeneous distribution of cells. Impedance was measured every minute at 41.5 kHz for the entire duration of cell culture, by the exposure of cells to small electrical currents delivered by electrodes on the plate surface. Cells were kept at 37°C and 5% CO2 inside the Maestro Edge throughout the experiment for impedance recording. Impedance data (resistance in ohms) was obtained with the AxIS Z software. Cells were grown in Skeletal Muscle Cell growth medium (SGM, PeloBiotech) and differentiated. First in basic differentiation media (bDM) and then in complete differentiation media (cDM), which includes different growth factors and extracellular matrix proteins to promote high myotube maturation (Toral-Ojeda et al., 2018, Lasa-Elgarresta et al., 2022).

#### Jess Western blot

RIPA lysis buffer (50 mM Tris-HCl pH 7.2, NaCl 0.9%, NP40 1%, EGTA 1 mM, EDTA 1 mM) with proteinase and phosphatase inhibitor cocktails (Thermo Fisher Scientific) and Cell-permeable inhibitor of calpain I, calpain II, cathepsin B and cathepsin L (Merck) was used to extract proteins. A Bovine Serum Albumin (BSA) concentration curve was used to quantify protein. Reagents and equipment for Jess Western blotting were all purchased from Protein Simple. Cell lysates were diluted at a final concentration of 0.5ug/ul with 0.1X sample buffer. 5X Fluorescent Master Mix was added to each sample at a 4:1 ratio (final concentrations of 1% (v/v) SDS and 40 mM DTT) and samples were incubated at 95°C for 5 min 3 mL of each sample were loaded into the cartridge. Subsequent rows of the plate were filled with blocking buffer (antibody diluent 2), primary and secondary antibody solutions, chemiluminescence reagents, and wash buffer according to the manufacturer’s instructions. Previously optimized primary antibodies were diluted in antibody diluent at different ratios (1:10 MyoD Antibody (C-20): sc-304, 1:10 sc-12732 - myogenin (F5D), 1:10 Myf-5 (C-20): sc-302), followed by HRP-conjugated secondary antibodies. Finally, the plate was spun down for 5 min at 1000 × g. Plates and capillaries were loaded into a Jess machine, and assays were carried out using the standard 12- to 230-kDa or 66-440 kDa separation range protocol. Compass reports data as spectra of chemiluminescence signals versus apparent MW by assigning ladder peaks to capillary positions. Peak area calculations were performed using the Gaussian method.

#### Fluorescence *in situ* hybridization (FISH) and immunocytochemistry (ICC)

ICC was performed on fixed and permeabilized cell coverslips. They were blocked (PBS Triton 0.1% with 1% BSA and 1% horse serum) and incubated with anti-MBNL1 (1:200, MB1a(4A8), DSHB) or anti-desmin (1:50, D33, Abcam) overnight at 4°C. Next, the coverslips were washed with PBS-T and cells were then incubated with biotinylated horse anti-mouse-IgG (1:150, Vector) for 1 h at RT. Elite ABC kit (VECTASTAIN) was used for 30′ at RT to amplify the signal, followed by some PBS-T washes and a 2-h incubation at RT with streptavidin-FITC (1:200, Vector). In the anti-MBNL1 incubated cells we subsequently performed FISH. For that, the cells were washed and incubated with acetylation buffer (1.16% triethanolamine, 0.25% acetic anhydride) for 10 m at RT. After pre-hybridization (SSC2X, 30% formamide) the cells were incubated with 1 μM Cy3-labelled (CAG)10 probe diluted 1:100 in hybridization buffer (40% formamide, 2× SSC, 0.2% BSA, dextransulfate 100 mg/mL, vanadyl 2 Mm, tRNA 1ug/mL, herring sperm 1 mg/mL) for 2 h at 37°C. Finally, the coverslips were washed and mounted in slides with Diamond Anti-Fade mounting medium with DAPI (Thermo Fisher Scientific). Images were taken with Zeiss AxioObserver Z1 microscope at 63× and analyzed with ZEN blue software and ImageJ.

#### Splicing analysis

Total RNA from primary and immortalized myotube cultures was extracted with the PureLink RNA Mini Kit (Invitrogen) according to the manufacturer’s instructions. 500 ng of RNA was retrotranscribed to cDNA using SuperScript IV Reverse Transcriptase (Invitrogen) according to the manufacturer’s protocol. One microliter of cDNA was used for the subsequent PCRs to analyze splicing alterations. Primers and PCR conditions are described in Table S2.

#### Treatment

To study treatment effect in the immortalized cell lines, we differentiated both primary and immortalized myoblasts into myotubes. On differentiation day 7 we added an antisense oligonucleotide (ASO), targeting the expansion repeat, for 48 h. The ASO used was a BNA^NC^ gapmer with the sequence AGCagcagcagCAG (Bio-Synthesis) in which capital letters mean BNA^NC^ modifications. The ASO concentration used was 30 nM.^48^ Transfection was performed in differentiation media containing 0.2% lipofectamine 2000 (Thermo Fisher Scientific) and 25% Opti-Mem (Gibco).

### Quantification and statistical analysis

#### Statistical analysis

Statistical analysis was performed with GraphPad Prism 8 software. Normality was determined with ShapiroWilk test. T-test or Mann-Whitney test were used for two-group comparison analysis while one-way ANOVA with Dunn’s post-test or Kruskal-Wallis test was used for comparison analysis between the three cell lines. ∗*p* ≤ 0.05, ∗∗*p* ≤ 0.01, ∗∗∗*p* ≤ 0.001, ∗∗∗∗*p* ≤ 0.0001.

## Supporting Citations

The following references appear in the supplemental information: ^80^^,^^81^^,^^82^.

## Supplemental information

Document S1. Figures S1‒S3, Tables S1, and S2
