# Immortalized human myotonic dystrophy type 1 muscle cell lines to address patient heterogeneity

**DOI:** 10.1016/j.isci.2024.111499

**Published:** 2024-12-09

**Authors:** Judit Núñez-Manchón, Júlia Capó, Alicia Martínez-Piñeiro, Eduard Juanola, Jovan Pesovic, Laura Mosqueira-Martín, Klaudia González-Imaz, Pau Maestre-Mora, Renato Odria, Estefania Cerro-Herreros, Neia Naldaiz-Gastesi, Adolfo López de Munain, Rubén Artero, Dusanka Savic-Pavicevic, Ainara Vallejo-Illarramendi, Kamel Mamchaoui, Anne Bigot, Vincent Mouly, Mònica Suelves, Gisela Nogales-Gadea

## Main text

(iScience *27*, 109930; June 21, 2024)

In the originally published version of this manuscript, four co-authors who had contributed significantly to the execution and interpretation of the reported study—Estefania Cerro-Herreros, Neia Naldaiz-Gastesi, Adolfo López de Munain, and Rubén Artero—were not included in the author list. This error was introduced during the preparation of the original version submitted. The article has now been updated to include the correct list of authors and affiliations and the updated authors contributions section. This correction does not affect the conclusions presented in the published article.

