# Supplementary material for: Immortalized human myotonic dystrophy type 1 muscle cell lines to address patient heterogeneity
Source: iScience. 2024 May 7;27(6):109930. doi: 10.1016/j.isci.2024.109930 (PMC11144749; doi:10.1016/j.isci.2024.109930)
Supplement: Document S1. Figures S1‒S3, Tables S1, and S2 [file mmc1.pdf]

iScience, Volume *27*

Supplemental information

Immortalized human myotonic dystrophy type 1

muscle cell lines to address patient heterogeneity

Judit Núñez-Manchón, Júlia Capó, Alicia Martínez-Piñeiro, Eduard Juanola, Jovan Pesovic, Laura Mosqueira-Martín, Klaudia González-Imaz, Pau Maestre-Mora, Renato Odria, Estefania Cerro-Herreros, Neia Naldaiz-Gastesi, Adolfo López de Munain, Rubén Artero, Dusanka Savic-Pavicevic, Ainara Vallejo-Illarramendi, Kamel Mamchaoui, Anne Bigot, Vincent Mouly, Mònica Suelves, and Gisela Nogales-Gadea

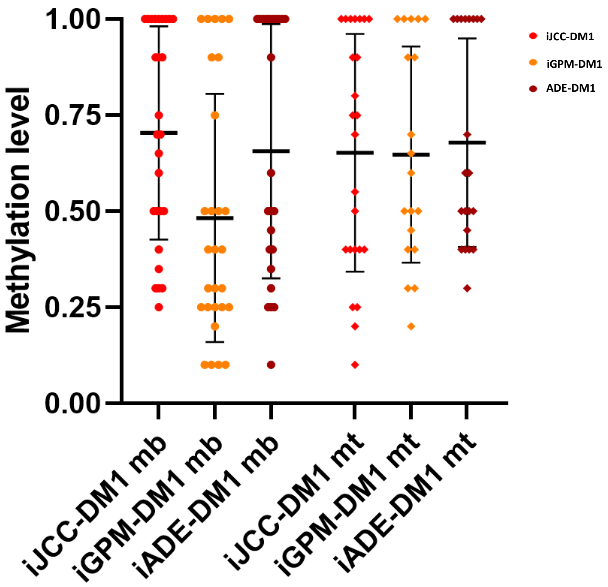

Supplementary Figure 1. Methylation levels did not differ between the three DM1 cell lines except for iJCC-DM1 vs iGPM-DM1 immortalized myoblasts. Related to Figure 2. "i" before cell line name means immortalized. "mb" and "mt" after cell line name mean myoblasts and myotubes, respectively. For each patient there are between 19 and 28 single measurements.

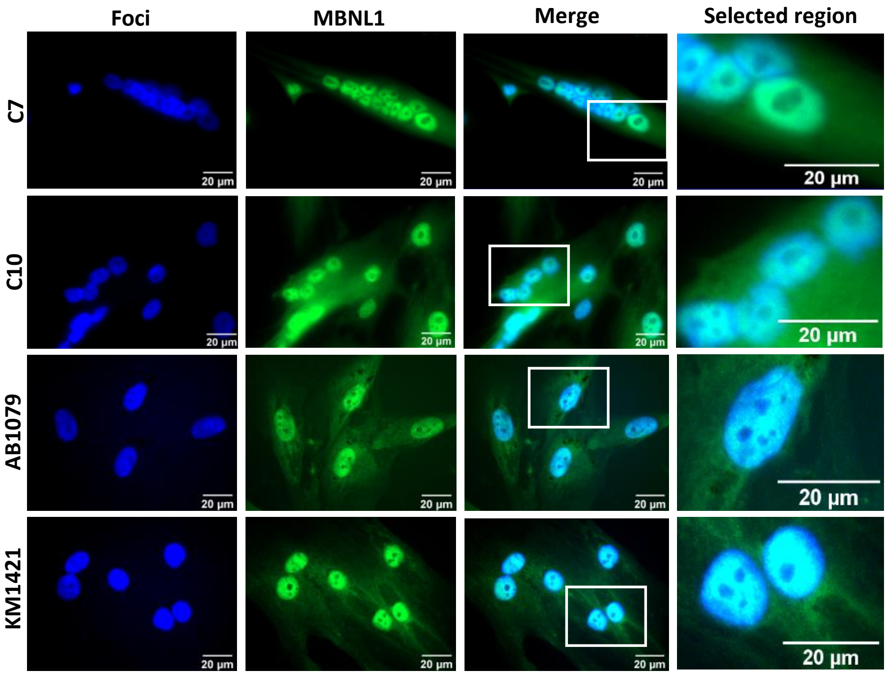

Supplementary Figure 2. Immortalized control myotubes do not show neither RNA foci or MBNL1 aggregates. Related to Figure 5. Foci (red), MBNL1 (green) and nuclei (blue) immunofluorescence analysis performed in 5 days differentiated primary and immortalized control myotubes.

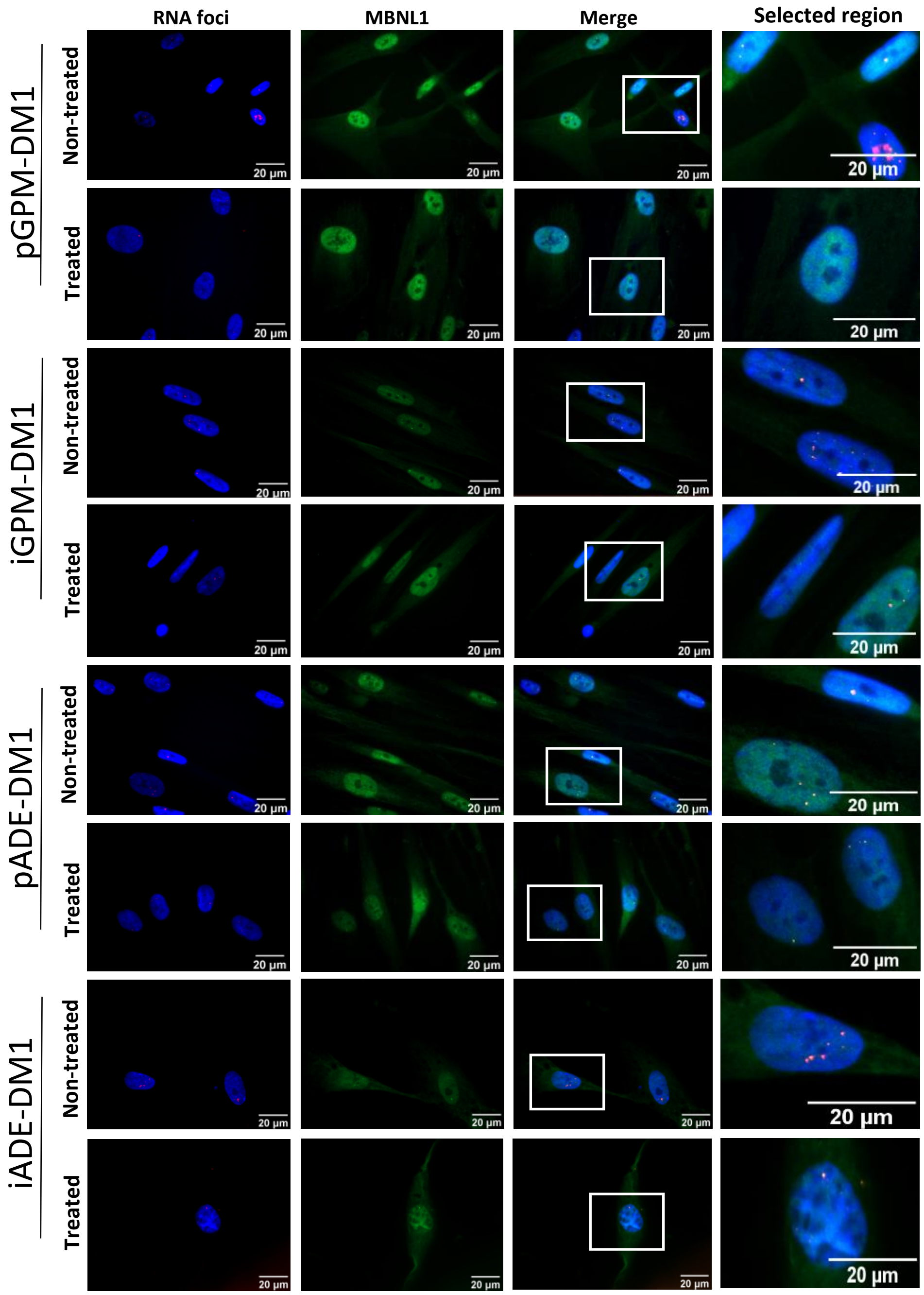

Supplementary Figure 3. Immortalized DM1 myotubes respond to treatment in a similar way to primary DM1 myotubes. Related to Figure 8. Foci (red), MBNL1 (green) and nuclei (blue) immunofluorescence analysis performed in 5 days differentiated primary and immortalized DM1 myotubes. “p” and “i” before cell line name mean primary and immortalized, respectively.

Supplementary table 1. Primers used for qPCR. Related to the STAR Methods

PCRs Transcript	Forward primer	Reverse primer	Fragment size (bp)
SIX5	AGGTCAGCAACTGGTTCAAG	ACTCGTCCTCAGTCGTGG	108
DMWD	GCTCTATTTCTACCCAGGCTG	TGAAATCGTGGCAGGTGG	113
DMPK	GCCCAGGACAAGTACGTG	CTTCATCTTCACTACCGCTACC	150
HPRT	TGAAATCGTGGCAGGTGG	ACAGAGGGCTACAATGTGATG	115

Supplementary table 2. Primers and conditions used for splicing analysis. Related to the STAR Methods

PCRs Transcript	Forward primer	Reverse primer	Annealing Tª	Cycles	Fragment sizes (bp)	Reference
***BIN1* (exon 11)**	AGAACCTCAATGATGTGCTGG	TCGTGTTGACTCTGATCTCGG	58	28	163-208	Fugier et al. 2011[32]
**MBNL1 (exon 7)**	GCCCAATACCAGGTCAACCA	GGCCTCTTTGGTAATGGGGG	58	35	101-155	Ketley et al. 2020[80]
***LDB3* (exon 11)**	GCAAGACCCTGATGAAGAAGCTC	GACAGAAGGCCGGATGCTG	61	28	163-352	François et.al 2011[81]
***INSR* (exon 11)**	CCAAAGACAGACTCTCAGAT	AACATCGCCAAGGGACCTGC	60	35	131-167	Savkur et al. 2011[31]
**DMD (exon 78)**	TTAGAGGAGGTGATGGAGCA	GATACTAAGGACTCCATCGC	58	28	116-148	Rau et. Al. 2015[28]
***ATP2A1* (exon 22)**	CTCATGGTCCTCAAGATCTCAC	AGCTCTGCCTGAAGATGTGTCAC	58	35	161-203	Wang et al. 2018[82]
KIF13A (exon 32)	TCCTGTCAGTATCCATCGGCT	TGAGTGCATCTGACCACCTCT	65	30	117-156	-
